# A Revised Perspective of Skeletal Stem Cell Biology

**DOI:** 10.3389/fcell.2019.00189

**Published:** 2019-09-13

**Authors:** Thomas H. Ambrosi, Michael T. Longaker, Charles K. F. Chan

**Affiliations:** ^1^Institute for Stem Cell Biology and Regenerative Medicine, Stanford University School of Medicine, Stanford, CA, United States; ^2^Hagey Laboratory for Pediatric Regenerative Medicine, Department of Surgery, Stanford University School of Medicine, Stanford, CA, United States

**Keywords:** bone, skeletal stem cell, bone marrow/mesenchymal stromal/stem cell, stem cell niche, heterogeneity, regeneration, aging

## Abstract

Bone-related maladies are a major health burden on modern society. Loss of skeletal integrity and regeneration capacity through aging, obesity, and disease follows from a detrimental shift in bone formation and resorption dynamics. Targeting tissue-resident adult stem cells offers a potentially innovative paradigm in the development of therapeutic strategies against organ dysfunction. While the essential role of skeletal stem cells (SSCs) for development, growth, and maintenance of the skeleton has been generally established, a common consensus on the exact identity and definition of a pure *bona fide* SSC population remains elusive. The controversies stem from conflicting results between different approaches and criteria for isolation, detection, and functional evaluation; along with the interchangeable usage of the terms SSC and “mesenchymal stromal/stem cell (MSC)”. A great number of prospective bone-forming stem cell populations have been reported with various characteristic markers, often describing overlapping cell populations with widely unexplored heterogeneity, species specificity, and distribution at distinct skeletal sites, bone regions, and microenvironments, thereby creating confusion that may complicate future advances in the field. In this review, we examine the state-of-the-art knowledge of SSC biology and try to establish a common ground for the definition and terminology of specific bone-resident stem cells. We also discuss recent advances in the identification of highly purified SSCs, which will allow detailed interrogation of SSC diversity and regulation at the single-cell level.

## Introduction

The skeleton is a composite of diverse tissue types, including cells of osseous, adipogenic, cartilaginous, stromal/endothelial, and hematopoietic lineages. Beyond its central role in locomotion, it has also been established as an endocrine organ that is essential for mineral homeostasis and systemic health (Karsenty and Ferron, 2012). Bone is fundamental for structural support and movement, which is reflected in the observation that people suffering from impaired bone fitness inevitably experience a decline in quality of life and increased morbidity (Demontiero et al., 2012; Lee et al., 2014). There is a growing focus on skeletal health worldwide due to the rising number of elderly people, which has dramatically increased the burden of bone fractures and skeletal disorders. One of the main metabolic diseases, which has expanded exponentially alongside that development, is osteoporosis, a condition resulting from an imbalance of the ability to form bone at the same speed as it is resorbed. Osteoporosis leads to increased fragility and occurrence of fractures (Pagnotti et al., 2019). There are over six million fractures per year in the United States alone, with the majority occurring in the older population. While in healthy individuals bones normally have the ability to heal without scarring, in up to ten percent of the cases fractures do not heal in an adequate time frame and develop to so-called nonunions, with limited treatment options (Ekegren et al., 2018). Along with osteoporosis, degenerative joint disease, or osteoarthritis, is a top cause of disability in the aged adult, which is characterized by deteriorated cartilage resulting in bone-to-bone rubbing creating increased stiffness, pain, and impaired movement. It is currently unclear why adult cartilage does not naturally regenerate like bone tissue. Importantly, osteoporosis and osteoarthritis are not only associated with aging but also sedentary lifestyle and metabolic health, underlining the systemic interrelation between bone health and physiological health (Chen et al., 2017). Therefore, advances in understanding of the stem and progenitor cells of bone and cartilage could have major ramifications for global health.

The cellular origins of bone and cartilage have been extensively studied. Like all connective tissues, the bone compartment contains cell populations with multipotent differentiation potential, generally referred to as “mesenchymal stromal/stem cells” (MSCs). Beside putative roles in controlling the bone marrow niche, these cells are thought to be the main source of bone formation during organ growth, maintenance, and repair. Given their beneficial properties, differentiation capacity, and expandability, strong efforts have been made to exploit those characteristics for cell-based regenerative therapies. The success of “MSC”-based approaches have been hampered by the high heterogeneity of the transplanted cell populations, mainly attributable to their varying tissue source but also to discrepancies in the techniques for detection, purification, and isolation of prospective stem cells (Galipeau and Sensebe, 2018). Research in mice is continually adding to the number of potential bone stem cell populations defined by cell surface proteins and genetic labeling, however, the majority of reports are limited by concerns of cell purity, lack information on the degree of overlap with previously reported cell populations, and most importantly are inappropriate for translation to the human setting (Bianco and Robey, 2015). A major drawback is that the identity and use of human bone marrow-derived stem cells have long been based on plastic culture adherence. Unfortunately, the relative technical ease of this technique and its use for isolating other morphologically similar fibroblastic cell types from various connective tissues coupled to deceptive *in vitro* differentiation regiments has helped fuel doubtful claims, offering “MSC cell therapies” for regenerative purposes, resulting in detrimental rather than beneficial outcomes (Sipp et al., 2018). First and foremost, there is no scientific rationale, or much less pre-clinical data, justifying the use of those cells from any tissue source for clinical application. Considering the extensive literature on bone-residing stem cells, there is a need for a more standardized functional characterization of potential cell types. Reported “MSCs,” or rather multipotent bone marrow stromal cell (BMSC) populations, display a variety of differences including developmental occurrence (e.g., pre- vs. post-natal), localization, and differentiation potential, with the most striking differences being obvious between classical perisinusoidal and growth plate/periosteal bone-forming cells, which will be discussed in detail (Sacchetti et al., 2007; Tormin et al., 2011; Chan et al., 2015, 2018; Ambrosi et al., 2017). Accumulating evidence suggests that the terms “MSC”/BMSC and skeletal stem cell (SSC), which have been used interchangeably, are describing both distinct and overlapping stem cell population with different properties and functions.

In light of these observations, this review aims to collectively compare reported bone-residing stem cell populations in mice and humans; and to establish a common terminology in order to promote a better basis for the development of successful research strategies. We have focused on findings of the appendicular skeleton, as the majority of scientific reports are based on experiments using limb and hip bone tissues. This is likely assignable to the ready access of specimen for these skeletal sites in mice and humans. It remains to be shown if findings can be generalized to all bone compartments and future investigations will have to explore if embryonic origin, skeletal form, and cell composition affect the SSC source. Importantly, existing controversies in the field are due to laboratory-specific availability as well as preference of technology and genetic models for the identification of “MSCs”/SSCs. Establishing a common ground will have great importance for a better understanding of scientific data and more efficient paradigms of regenerative approaches.

## Defining Skeletal Stem Cells

Stem cells are characterized by their ability to self-renew and to differentiate into multiple cell fates thereby contributing to tissue ontogeny, growth, and turnover for regeneration throughout life (Bianco and Robey, 2015). All cells of an organism are descendants of a zygote with unique totipotency, which is lost after the preimplantation stage of the blastocyst, with exception of germline stem cells (Evans and Kaufman, 1981; Martin, 1981). At that timepoint, defined multipotent, fate-restricted fetal stem cells (and then postnatal stem cells) emerge, orchestrating organ maturation and maintenance. It has to be stressed that despite some early controversial claims there is no evidence for the existence of stem cells with *in vivo* pluripotency in adult tissue (Jiang et al., 2002; Miyanishi et al., 2013). However, ground-breaking advancements in cellular reprograming have been able to generate induced pluripotent stem cells from diverse somatic cell origins *in vitro* (Takahashi and Yamanaka, 2006).

The concept of stem cells dates back as far as the middle of the 19th century, when Ernst Haeckel first coined the term “Stammzelle” (Dose, 1981), suggesting the origin of living cells as an evolutionary sequence. This theory was extended and experimentally addressed by contributions of pioneers including Arthur Pappenheim and Alexander Maximov, eventually leading to the seminal finding of the existence of a hematopoietic stem cell (HSCs) by Till and McCulloch, as they described that single rare bone marrow cells could form multilineage myelo-erythroid colonies in the spleen of lethally irradiated mice (Till and McCulloch, 1961; Becker et al., 1963). This discovery provided the first definitive proof of the presence of a postnatal *bona fide* stem cell but did not yet enable the prospective isolation of phenotypically defined cells. With the development of more sophisticated technologies such as flow cytometry, a cell population considerably enriched for HSCs was later first described by Spangrude et al. (1988) building the foundation for today’s concept of the hematopoietic lineage tree (Laurenti and Gottgens, 2018).

Recent efforts have led to the identification of multiple types of lineage-restricted postnatal stem cells with self-renewal and multipotent differentiation ability in many other organs. The study of stem cells has given important insights into mechanisms of tissue biology that provide great potential for the development of novel cell-based regenerative therapies. However, the exciting nature of stem cell concepts has also promoted the spread of dubious physicians and companies offering unproven stem cell-based therapies that lack peer-reviewed demonstrations of their efficacy as well as IRB (Internal Review Board)- and FDA (Federal Drug Administration)-type approvals, leading to adverse outcomes and harming the reputation of the stem cell research field (Sipp et al., 2018). One of the main causes is the incorrect use of the term “stem cell” for weakly defined cell populations comprised of heterogeneous cell types, i.e., more committed cell populations with varying differentiating potential and cells from other lineages. A striking example can be found in the field of bone marrow transplantations, which are often advertised as “hematopoietic stem cell transplants” but which in actuality are relatively unenriched mixtures of many cell types with only a very small number of stem cells. Some of the cell types that are co-transplanted include lymphocytes that could cause graft vs. host disease (GVHD), though in some cases they could also lead to helpful graft vs. tumor responses (Szyska and Na, 2016). Transplanting impure, undefined mixtures of cells not only hampers the predictability and reproducibility of treatments but may additionally lead to false assumptions by patients and disappointing results that could tarnish popular perceptions of stem cell research and stem cell-based medicine. It is, therefore, of great importance to re-establish solid foundations for stem cell research that focuses on highly purified, homogeneous populations and uses both, *in vitro* and *in vivo* assays, to identify true stem cells that display all the hallmark stem cell characteristics, i.e., quiescence, self-renewal, and multipotency at the single-cell level (Weissman, 2002; Bianco et al., 2008).

### The Road to Identifying the Skeletal Stem Cell

Almost paralleling the description of the HSC, evidence for the existence of a second bone-resident stem cell appeared. This type of stem cell was distinct from HSCs as it was proposed to be the origin of non-hematopoietic stroma. Groundbreaking experiments by Tavassoli and Crosby (1968) in the 1960s reported that boneless marrow pieces have the capacity to reconstitute hematopoietic and adventitial structures, complete with bone, cartilage, and stromal tissue, providing a newly formed microenvironment for functional hematopoiesis. Friedenstein was one of the first to assign osteogenic potential to non-hematopoietic plastic-adherent cells with fibroblast colony-forming unit (CFU-F) capacity and multi-differentiation ability upon *in vivo* transplantation (Friedenstein et al., 1966; Friedenstein et al., 1974; Castro-Malaspina et al., 1980; Kuznetsov et al., 1997). It took another decade before Owen and Friedenstein (1988) proposed the concept of clonogenic, multipotent, self-renewing stromal cells *in vivo*, which were shortly after defined as “Mesenchymal Stem Cells” (Caplan, 1991; Pittenger et al., 1999). The International Society of Cellular Therapies (ISCT) later re-defined the same cells as “Mesenchymal Stromal Cells” since “stroma” (*Greek*: bed) better reflected the hematopoiesis-supportive function and heterogeneous mixture of cell types included in “MSCs” (Dominici et al., 2006). In contrast to HSCs, which were conclusively shown to be able to give rise to phenotypically defined cells reconstituting hematopoiesis under limiting dilution conditions in serial transplants, *in vivo* self-renewing capacity had not been convincingly proven for cells of mesenchymal origin in mice and humans for another substantial period of time (Sacchetti et al., 2007; Mendez-Ferrer et al., 2010). By developing a renal transplant assay for single skeletal progenitor cells, we could further test the *in vivo* self-renewal capabilities of bone-forming stem cells without prior CFU-F expansion (Chan et al., 2013). Perplexingly, and as a result of the relative ease of culturing plastic-adherent fibroblastic cells, non-skeletal “multipotent stromal cell” populations have incorrectly been defined by their ability to make bone, cartilage, fat, tendon, and muscle in the culture dish, sometimes even suggested to be pluripotent depending on the assay conducted (Via et al., 2012).

While mostly associated with bone and adipose tissue, “MSC”-like populations have been identified in other connective tissues as well as perinatal tissues such as the umbilical cord and the placenta. As a consequence, the denomination “MSC” was established as a universal term for adult tissue-resident stem cells with putative therapeutic promise and is up to today routinely used by many scientists to wrongly describe stem cell populations (da Silva Meirelles et al., 2006). The general use of the term “Mesenchymal Stem Cell” is flawed in many ways leading us to stress that its use should be avoided. The intrinsic properties of these cells are tightly correlated with their tissue source. They contain cells of divergent developmental origins and as mostly looked from the perspective of adult stem cells should not be named “mesenchymal” in the first place, since this term should be reserved for embryonic tissue. Above all, “MSCs” describe a highly heterogeneous cell population with seemingly multilineage differentiation capacity which actually results from the aggregate potential of a mixture of individual, distinct cell types. Over time, the promiscuous use of the “MSC” terminology resulted in a great number of publications with conflicting claims and it became necessary for the scientific community to address the inconsistencies. In an attempt to unify researchers and to rectify falsely assumed stem cell characteristics the ISCT published minimal criteria for the definition of “MSCs” and proposed to call them “Multipotent Mesenchymal Stromal Cells,” rather than “Mesenchymal Stem Cells” (Dominici et al., 2006). However, it should be noted that even “stromal” is an inaccurate generalization as it does not apply to fibroblasts of certain tissue sources, e.g., umbilical cord and amniotic fluid. Minimal criteria for human “MSCs” included plastic-adherence under standard culture conditions, which upon culture express CD105, CD73, CD90 and lack CD45, CD34, CD14 or CD11b, CD79α or CD19 and HLA-DR surface molecules, and exhibit tri-lineage differentiation potential into osteogenic, chondrogenic, and adipogenic fates *in vitro*. It needs to be stated that these cell surface markers are not specific for stem cells but rather confirm the fibroblastic identity of cultured cells. As a consequence, many laboratories are currently using widely differing marker profiles additionally stemming from differences in marker expression on freshly isolated cell populations. *In vitro* culture alters surface marker profiles and does not reflect the true biological activity of investigated cells, underscoring the need for the ISCT standards to be reassessed. Lastly, stemness of a homogeneous cell population is often not rigorously interrogated which in part could be attributed to the lack of clonal self-renewal as one defining criteria in the guidelines agreed on by the ISCT, in turn hampering success of clinical applications utilizing stromal cell populations (Robey, 2017). The FDA has published a study that describes the disagreement over molecular characteristics of “MSCs” (Mendicino et al., 2014), which is also supported by the report that identical surface protein profiles between stromal cell populations from different source tissues display differential transcriptional expression patterns (Sacchetti et al., 2016). Caplan, who originally coined “MSCs,” recently suggested refraining from the use of the word “stem cells” considering the issue of cellular heterogeneity and the inconclusive reports that are prevalent in “MSC”-related literature (Caplan, 2017).

The recent identification of *bona fide* lineage-restricted SSCs within the skeletal compartment, which give rise to a hematopoiesis-like hierarchy of committed progenitors *in vivo*, supported the need to re-think the nomenclature of skeletal derived “mesenchymal” stem cell-like cells (Sacchetti et al., 2007; Chan et al., 2015, 2018). In light of these developments, the terms “Bone marrow stromal cells” (BMSCs) and “Skeletal stem cells” (SSCs) have been suggested (Bianco and Robey, 2015). Further adding to the panoply of previously characterized skeletal progenitors are recent experimental findings that imply the existence of more than one *bona fide* SSC population, which will be discussed in the following sections.

### Getting a Grasp on SSC Terminology

An overwhelming number of researchers use the terms SSC and “MSC” interchangeably to describe bone-residing “stem” cell types, while actually reporting different or only partially overlapping skeletogenic populations without testing for basic stem cell characteristics, such as self-renewal. On the background of earlier mentioned discrepancies in the field, one should carefully distinguish SSCs as cells locally restricted to bone with the ability to reconstitute an environment for active hematopoiesis, as being able to self-renew on the clonal level, and to show multipotency within the same clone under *in vivo* conditions (Bianco et al., 2008). “MSCs” on the other hand, represent a heterogeneous cell mixture roughly complying with ISCT’s minimal criteria that can be isolated from tissues such as muscle, fat, bone, and heart, thereby proposing equal suitability for regeneration across connective tissues. While these stromal cell populations may genuinely contain some tissue-specific stem cells capable of generating tissue fates of their native tissue origin (Sacchetti et al., 2016), it is unlikely that they are intrinsically pluripotent. For example, BMSCs are inherently osteochondrogenic, muscle “MSCs” are myogenic, and cord blood-derived cells display spontaneous chondrogenesis *in vivo*. Functionally, categorization of SSCs vs. “MSCs” has been attempted by stemness assays (*in vivo* vs. *in vitro*), mode of action (regeneration vs. cues), therapeutic potential (regeneration by living cells vs. paracrine modulation/anti-inflammatory signaling), and type of administration (transplantation for engraftment vs. infusion for embolization) (Bianco et al., 2013), albeit with limited success.

On the basis of immune-localization studies with stromal cell markers that are also associated with blood vessels in different tissues (Crisan et al., 2008; Sacchetti et al., 2016), some stromal cell populations have further been described to be pericytes (Sacchetti et al., 2007; Caplan, 2008). In contrast, SSCs are enriched in and around the avascular regions of the hypertrophic growth plates, though they have also been found in the periosteum as well as fracture calluses (Chan et al., 2015, 2018; Debnath et al., 2018; Duchamp de Lageneste et al., 2018). It is, therefore, possible that BMSCs are either comprised of more committed progenitor populations descending from SSCs settling in sinusoidal regions or contain a multipotent pericyte-like stem cell giving rise to more restricted progenitor cell types itself. While neither scenario can be excluded, a combination of both might be an additional alternative explanation but remains to be investigated. An argument supporting this theory is the fact that we and others have found SSCs to be restricted to give rise to bone, cartilage, and stroma, but not fat *in vivo* (Chan et al., 2013, 2015, 2018; Mizuhashi et al., 2018). The CD146-positive SSC population identified by Sacchetti et al. (2007) does not overlap with our described SSC, which is in compliance with its adipogenic potential and subendothelial localization. However, we have also shown that a distinct lineage of CD146-positive multilineage skeletal progenitors is derived from CD146-negative SSCs (Chan et al., 2018). Additionally to osteochondrogenic mouse SSCs, an adipogenic lineage with phenotypically defined subpopulations sharing a common “mesenchymal” stem cell-like origin has been reported, further supporting the potential existence of two distinct SSC populations (Chan et al., 2015; Ambrosi et al., 2017). The collaboration of multiple stem and progenitor cell types in orchestrating the highly complex cell network within the bone compartment seems more reasonable given the manifold niches existing in the skeleton. At this point, however, we cannot definitively rule out that divergent findings might be the result of different experimental techniques, a topic that will be discussed in depth in sections below. Should it turn out to be confirmed, it could pose a great opportunity to unite the scientific community in agreeing on a common nomenclature which could accommodate the existence of two or more *bona fide* SSC populations in bone.

## Prospective Isolation of Skeletal Stem Cells

Over time, stem cell hallmark criteria have been extended by single and combinatorial use of surface marker/genetic labeling, transplantation assays, and *in vivo* lineage tracing. This has led to the discovery of multiple SSC populations in the growth plate, the endosteal and perisinusoidal bone marrow space, or the perichondrium. Nevertheless, the description of these cell populations is often incomplete or redundant leaving contradictory results that hamper the advancement of the field. A potential explanation can be found in the complex and plastic nature of bone tissues and might be vested in technical limitations or an indication for the presence of an everchanging network of multiple subtypes of SSCs orchestrating skeletal homeostasis and repair.

### Human Bones

Until the invention of flow cytometry by the Herzenbergs (Dangl and Lanier, 2013), which enables high-throughput, prospective isolation of antibody-labeled and fluorescence-tagged cell types, conclusions about putative SSC populations could only be drawn retrospectively leaving reasonable doubt about their true identity. Taking advantage of this technology, the CD45-negative non-hematopoietic nature of fibroblast colony-forming cells has been confirmed and copious markers, including CD105 (ENG), CD140a (PDGFRα), CD73 (NT5E), CD90 (THY1), STRO-1 (HSC70;HSPA8), CD271 (NGFR), and CD44 have been proposed to broadly label human SSC populations with variable CFU-F ability and differentiation to osteogenic, chondrogenic, adipogenic, and stromal lineages (Shi and Gronthos, 2003; Lange et al., 2005; Sorrentino et al., 2008; Pinho et al., 2013). CD271 (/CD140a^–/low^) and STRO-1 turned out to most efficiently select for perivascular residing SSC-like cells that are also able to maintain human HSCs for extended time in culture but are less restricting than CD146 (MCAM), which allegedly identified the entire CFU-forming fraction (Simmons and Torok-Storb, 1991; Sacchetti et al., 2007; Li et al., 2014). Single cell-derived colonies of CD45^–^CD146^+^ cells are sufficient to recreate hematopoiesis-supportive human ossicles in a mouse, representing an enriched SSC population with serially transplantability, multipotency, self-renewal, and localization along marrow sinusoids expressing endothelial and hematopoiesis supporting factors. However, a later paper by Tormin et al. (2011) reported at least similar CFU-F ability using CD271^+^CD145^–/low^ compared to CD271^+^ CD146^+^ cells. This is in line with our recently identified human SSC which falls into the CD146-negative fraction and underscores the necessity to enrich single marker labeled cell population with additional markers to derive homogeneous cell populations more depictive of the complex composition of skeletal tissue (Chan et al., 2018).

Assigning specific marker profiles to plated SSC populations is a major pitfall as *ex vivo* culturing alters the endogenous expression of surface markers. Cell epitope expression can be artificially skewed as seen with the loss of CD271 expression in cultured bone marrow cells in media containing basic Fibroblast Growth Factor (bFGF), as well as with CD146 which possesses a serum response element in its promoter and is enhanced in stromal cells under standard culture conditions and downregulated if exposed to a hypoxic environment (Tormin et al., 2011). Alike, CD44 is expressed by cultured “MSCs” only (Qian et al., 2012). Notably, simple variations in cell-detaching methods and culture media before flow cytometric analysis can alter cell surface antigen expression (Hagmann et al., 2013; Tsuji et al., 2017). The same thing might hold true for the digestion of freshly harvested tissue routinely conducted for FACS preparations which can have profound effects on the integrity of surface molecule expression and additionally is prone to loss of more fragile cell types (Autengruber et al., 2012). Altogether, this might under- or overestimate the abundance of cell populations as well as likely contaminate investigated candidate SSCs compromising conclusions drawn from experiments. It is, therefore, of the highest importance to confirm flow cytometric findings with *in situ* analysis of marker expression on tissue sections or by other methods.

In a meticulous endeavor, our laboratory was able to identify a highly purified *bona fide* SSC distinct from the reported CD146-positive SSC; and found in fetal and all adult stages throughout different skeletal sites but specifically enriched in the hypertrophic zones of the growth plate labeled by PDPN, CD73, CD164 and lacking expression of CD235, CD45, CD146, Tie2, and CD31 (Chan et al., 2018). We arrived at that surface marker panel by micro-dissecting seven regions of the long bones, looking for the enrichment of SSC markers found in the previously identified mouse SSC (Chan et al., 2015). Of note, in our analysis, CD146 was specifically enriched in diaphyseal regions abundant with vasculature separate from the growth plate – a site that is highly enriched with SSCs (Chan et al., 2018). We stringently tested *in vitro* and *in vivo* capacity of this candidate SSC population by serial transplantations, self-renewal, and lineage diversity to prove stemness. These human SSCs are activated by skeletal injuries, such as fractures, to locally increase proliferation and differentiation in order to facilitate regeneration. Importantly, we now have established a hierarchy with a SSC at the top giving rise to defined stromal subpopulations with osteogenic and/or chondrogenic fates; and the missing ability to give rise to fat. Although flow cytometric analyses identify phenotypic human SSCs at various skeletal compartments, future studies have to assess if cells from different bone types share functional properties with the investigated long bone-derived human SSCs. It is likely that bones with varying developmental origin do not share the same SSC and that transcriptional differences in SSCs from different skeletal sites determine skeletal shape and size.

**FIGURE 1:**
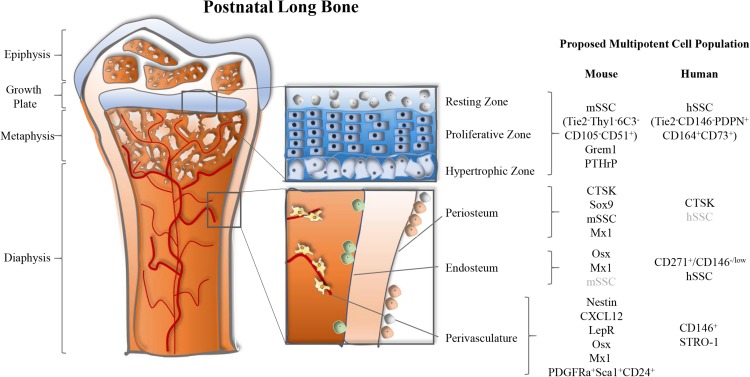
Multiple anatomical sites of long bone-resident skeletal stem and progenitor cell populations in mice. Postnatal long bones of mice harbor skeletal stem and progenitor cells at the growth plate, periosteum, endosteum, and perivascular sites. Several markers to identify these cell populations have been proposed and are summarized by location.

Taken together, the distinct cell marker expression (CD146^–^ vs. CD146^+^) of our and Sacchetti et al.’s (2007) SSC population, the divergent anatomical sites (epiphyseal vs. perivascular), and the disparity in lineage output (osteochondrogenic vs. osteochondroadipogenic) might be reconciled by our observation that CD146^–^ give rise to CD146^+^ multipotent skeletal progenitors. It might also be possible that the two cell types mark specific subsets of SSCs. This is further supported by the notion that CD146-negative cells were reported to migrate from bone marrow sinusoids to an endosteal niche (Tormin et al., 2011), a site we did not specifically investigate, but that would make sense given the role of SSCs in injury response (Figure 1). Intriguingly, independent observations in mouse bones add evidence for at least two SSC forms and will be discussed hereafter. Future studies will have to directly experimentally address this idea and will help to get more insight into potential functional differences between these two cell types.

### Mouse Bones

Mice provide an ideal model organism to research SSC biology. In contrast to humans, mice offer the accessibility of transgenic tools to specifically label and track cell populations beyond cell surface marker expression. Besides fluorescence reporter expression from an endogenous promoter of putative marker genes, the development of lineage tracing technology facilitated by the Cre-LoxP system has noticeably progressed the field, albeit with recognizable caveats (discussed later). Cre-LoxP recombination allows site-specific deletion, insertions, translocations, and inversions in the DNA of cells. This is facilitated by a single enzyme, called Cre-recombinase, which was originally derived from the bacteriophage P1 together with the LoxP site (Bouabe and Okkenhaug, 2013). Upon Cre-recombinase expression under the promoter of a target transgene, the enzyme acts on the second transgene containing the LoxP site. This leads to an irreversible gene sequence alteration which can be exploited in manifold ways, e.g., to continually turn on (fluorescence protein) or excise a gene (primary fluorescence protein or functional gene). Cre-reporter systems enable investigations in an unperturbed manner by either constitutive Cre-expression or time-point restricted induction through tamoxifen application in the modified CreERt version, being dependent on structural changes for nucleus translocation before eliciting recombinant properties.

Using this technology in combination with Rainbow-mice which carry an allele that through Cre-mediated recombination allows the stochastic expression of different fluorescence colors, we were able to show clonal expansion within the growth plate, conveying initial evidence for SSC activity in this region (Chan et al., 2015). This was preceded by reports of a purified cell population containing putative SSCs that started accumulating in the late 2000s when the Matsuzaki group first published a defined cell population with stem cell characteristics in mice. Establishing that stromal cells come from a distinct lineage compared to HSCs, the initial discovery found fibroblast activity within the PDGFRβ-positive cell fraction (Koide et al., 2007). They later narrowed down their findings to a perivascular population with *in vivo* self-renewal/multipotency to CD45^–^Ter119^–^Sca1^+^PDGFRα^+^ expressing cells, which *in vitro* contains clones with osteogenic, chondrogenic, adipogenic, and even angiogenic and neural crest differentiation ability (Morikawa et al., 2009a, b). They also suggested that the double-negative Sca1^–^PDGFRα^–^ cells were more restricted and committed osteoprogenitors. Many more surface proteins have been proposed to label multipotent clonogenic stromal cell types, partially mirroring reports in humans, e.g., CD271, CD90, CD51, CD44, CD146, CD106, and LepR with variations depending on the developmental stages investigated (Mabuchi et al., 2013; Isern et al., 2014; Zhou B.O. et al., 2014). Building on these studies and employing a surface marker configuration described for progenitor cell populations of adipose tissue (Rodeheffer et al., 2008), a bone-resident stem cell-like population that is CD45^–^CD31^–^Sca1^+^PDGFRα^+^CD24^+^ was isolated which shows high purity for clonal tri-lineage potential in *in vitro* studies and *in vivo* when transplanted in bulk (Ambrosi et al., 2017). In the bone marrow these cells give rise to committed adipogenic progenitors (CD24^–^) that further make Zfp423-expressing pre-adipocytes before becoming mature fat cells. In agreement with the observations by Morikawa et al. (2009b), an alternate differentiation route creates osteochondrogenic-restricted Sca1^–^PDGFRα^–^ cells. Our lab first described another subpopulation within the latter, defined by CD105 expression bringing along minimal properties for the generation of a hematopoietic niche through endochondral ossification and found in fetal, neonatal, and adult mouse bone (Chan et al., 2009, 2013). Using a renal capsule transplantation model, we unprecedently showed that single cells termed Bone Cartilage Stromal Progenitor (BCSPs; CD45^–^Ter119^–^Tie2^–^Thy1^–^6C3^–^CD51^+^) were *bona fide* stem cells *in vivo*, spawning stromal subsets crucial for niche functions (Chan et al., 2013). Hypothesizing that the skeletal lineage might follow an analogous differentiation tree as described for the hematopoietic compartment, we set out to delineate the SSC and its downstream stroma. We succeeded in showing that skeletogenesis proceeds through a developmental hierarchy of lineage-restricted progenitors with a *bona fide* SSC at the top (CD45^–^Ter119^–^Tie2^–^Thy1^–^6C3^–^CD51^+^CD105^–^CD200^+^) giving rise to the BCSP and seven defined and restricted stromal progenitor cell types, never producing fat (Chan et al., 2015; Gulati et al., 2018). In concordance with the different locations SSCs have been identified this raises the possibility that the perivascular adipocyte-producing multipotent stromal cells represent a distinct stem cell-like population, as also observed in experiments with human bone marrow cells. One major limitation of the multi-surface marker approach is the inability to investigate the exact niche composition and to trace cell fates through *in situ* labeling. Using transcriptomic data from highly purified cell populations representing all cell types of long bones might allow for the identification of a single specific marker for mouse SSCs which then can be used to generate transgenic mice for a more defined interrogation of their biological activity.

Concurrently, the generation and analysis of genetically modified mice have yielded enormous insights into skeletal biology. Using single fluorescence reporters, e.g., GFP, dtTomato, mCherry, YFP, etc., under the control of target gene expression, many cell populations have been described enriching for colony-forming fibroblasts, relating to widely overlapping cell populations termed SSCs, “MSCs,” pericytes, or CXCL12-abundant reticular (CAR) cells depending on the publication. Furthermore, genetic manipulation of stromal populations *in situ* employing the Cre-system has allowed investigations of skeletal development, homeostasis, and regeneration through temporal lineage tracing and targeted ablation of specific cell types. Cre-mediated recombination by the transcription co-activator Paired-related homebox 1 (Prx1) is very effective in inclusive labeling of all clonogenic stromal cells as it incorporates but is not limited to PDGFRα/β- and Sca1-expressing cells (Logan et al., 2002; Omatsu et al., 2010; Ambrosi et al., 2017). It also includes CAR cells, a very crude collection of stromal progenitor cells (PDGFRα-positive cells are a subpopulation), since it also strongly labels endothelial and mature osteogenic cells (Sugiyama et al., 2006; Greenbaum et al., 2013), which all play crucial roles for normal homeostatic processes of the skeleton, as shown by aberrant differentiation and hematopoietic support when specifically ablated (Omatsu et al., 2010; Seike et al., 2018).

One of the first fluorescent labels to be used for mouse BMSCs, initially reported as a marker for neural stem and progenitor cells and also expressing PDGFRα, is Nestin (Mignone et al., 2004). In the marrow, Nestin-GFP marks perivascular, clonogenic, self-renewing cells, which can form mesenspheres that differentiate in the osteochondral lineages *in vivo* as well as to adipocytes *in vitro* (Mendez-Ferrer et al., 2010). A subsequent demonstration by the same group showed that Nestin-expressing cells could be divided into Nestin^*dim*^ and Nestin^*bright*^ cells, with the cells having the brightest signal being NG2-positive, more quiescent, and containing the highest CFU-F potential while the dimly labeled cells overlap with LepR (Kunisaki et al., 2013). Surprisingly, no Nestin or NG2 gene expression can be found in Prx1-Cre labeled cells, an observation that has been explained to potentially be due to incomplete tracing (Greenbaum et al., 2013). The stem cell-like nature of Nestin-GFP cells has also been implied by their descent from Col2-expressing cells, however, although Col2 marks progenitor cell types it is rather ubiquitously expressed which make such conclusions questionable (Maes et al., 2010; Ono et al., 2014a). Furthermore, the Nestin gene has been utilized for lineage tracing through Cre-recombination which revealed varying proportions of endothelial cells that were marked depending on the model used, leaving considerable doubt with regard to its use as a proper stem cell marker (Ding et al., 2012; Ono et al., 2014a). Similarly, the heterogeneous Mx-1-Cre line overlaps with Nestin-GFP, PDGFRα, Sca1, CD105 but also robustly labels more mature osteogenic populations (Osterix^+^, Osteopontin^+^, Osteocalcin^+^) as well as hematopoietic cells (Park et al., 2012).

In part due to the vague labeling of stromal cells by Nestin, LepR-expressing cells (traced by Cre and CreERt) have emerged as the prevailing multipotent stromal cell marker over the last years (Ding et al., 2012; Zhou B.O. et al., 2014). In a series of elegant studies, the Morrison lab could show that LepR-expressing cells are the major source of osteogenic, chondrogenic, and adipogenic cells formed during adulthood and are involved in homeostasis, fracture healing, and hematopoiesis (Zhou B.O. et al., 2014; Yue et al., 2016). LepR-expression in the bone marrow overlaps with all known stromal cell markers to varying extents. If interpreting histological data of LepR-reporter mice bones, caution is advised as they also label substantial proportions of hematopoietic and endothelial cell types which can be excluded during FACS-purification. Just under 10% of LepR-labeled cells are CFU-F of which about the same percentage possesses *in vitro* clonogenic tri-lineage potential underlining the often wrongly used term SSC for these cells as they are not pure *bona fide* SSCs (Zhou B.O. et al., 2014). Recent single-cell RNA-sequencing reports of LepR-positive cells have suggested that this population contains at least four subpopulations with differing osteogenic and adipogenic commitments (Tikhonova et al., 2019). In accordance with that as well as our proposed skeletal lineage tree, David Scadden’s group also using single-cell RNA-sequencing identified seventeen marrow stromal subsets characterized by fibroblastic, endothelial, pro-adipogenic, and pro-osteochondrogenic phenotypes, of which all expressed LepR in at least some cells (Baryawno et al., 2019). Although these studies shed exciting new light on the abundant heterogeneity of single fluorescence-labeled cell populations, attention is warranted, as results are mostly descriptive, and assumptions are based on former knowledge. Future studies will have to provide evidence that stromal sub-populations identified by singe cell RNA-sequencing are following proposed differentiation trajectories *in vivo* and fulfill functions assigned based on gene expression.

LepR-expression can also be found in the mouse SSCs (mSSCs; plus, all downstream lineages) identified by us, which show a strong resemblance to BMP-antagonist Gremlin1-Cre traced cells (Worthley et al., 2015). Grem1^+^ cells predominantly reside in metaphyseal areas, show stem cell hallmark characteristics on the clonal level, and are non-adipogenic. However, neither Nestin-Cre nor LepR-Cre seem to substantially trace Grem1 cells, which on the one hand supports the notion of a concept with at least two skeletogenic stem cell populations; and on the other hand might be the result of the infidelity of these Cre-reporters, e.g., incomplete recombination, or the time of initiated tracing. Latest single-cell data is supportive of a stem cell model in which one SSC source is following a classic tri-lineage differentiation pathway that is complemented by a second origin for osteoblastic cells created from a “chondrogenic cluster” (Baryawno et al., 2019). The latter might entail the mSSC/Grem1^+^ cells as they are found within the boundaries of highly chondrocyte-abundant growth plate areas.

Along this chain of arguments, a recent publication describes parathyroid hormone-related protein (PTHrP) as a label for chondrocytes of the resting zone in the growth plate of long bones which descend from a PTHrP^+^ SSC. PTHrP indeed seems to enrich for mSSCs but remains heterogeneous for other more committed cell populations (Mizuhashi et al., 2018). These cells are shown to contribute to CAR cells and bone stroma (not fat) below the chondrogenic zones, even though only to a low degree. A potential explanation is the incomplete labeling of stem cells by the Cre-driver, also observed when trying to ablate PTHrP-expressing cells. Another study reported Glioma-associated oncogene 1 (Gli1) as a marker for early postnatal multipotent progenitor cells in the metaphyseal region of long bones (Shi et al., 2017). Shi et al. showed crucial functions for Gli1-cells in maintaining bone mass and the capacity to produce chondrocytes, osteocytes, and adipocytes *in vivo*. Experiments also confirmed a strong overlap with other BMSC markers such as PDGFRα/β and LepR. Combined with the observation of expression of markers usually seen in more mature cell types (i.e., Osx, Col1), this data implies that Gli1 enriches for multipotent stromal cells but does not uniquely mark a homogeneous SSC population. Future studies could more closely assess SSC content of Gli1-positive cells by clonal analysis, an approach that was not pursued in that work.

Aside from the association of SSCs at and around the growth plate, they are also abundant along the periosteum, where they can quickly get “activated” upon injury and facilitate proper fracture healing (Marecic et al., 2015). We discovered that these stem cells additionally express CD49f and display stronger proliferative and bone-forming capacity than their CD49f-negative counterparts. Colnot and colleagues confirmed the existence of a multipotent periosteal cell pool with high clonogenic as well as tri-lineage differentiation potential *in vitro* and *in vivo* that plays an important role in fracture regeneration (Duchamp de Lageneste et al., 2018). These cells were shown to be dependent on the expression of the extracellular matrix protein Periostin, as the loss of it impairs their number and function. A unique marker for SSCs of the periosteum has long remained elusive and could also not be provided by the latter study which relied on plastic adhering cells derived from micro-dissected periosteum and bone marrow cultured in media. Recently, however, Cathepsin K (CTSK), best known as an osteoclast marker, has been demonstrated to label a set of skeletal cells present on the periosteum (Debnath et al., 2018). Functional characterization of CTSK-Cre;mTmG reporter mice revealed general multipotent stromal cell characteristics and a role in fracture healing, but no involvement in hematopoiesis support. Looking ahead, the ability of periosteal SSCs (pSSCs) to be efficiently activated upon injury and their anatomical localization lining the bone surface makes them an attractive source and target for clinical treatments.

In sum, single-gene transgenic lineage tracing approaches have key limitations since many of the frequently used genes to trace SSC lineages, including PTHrP, Nestin, and LepR, are broadly expressed by multiple tissue types and are almost ubiquitously expressed within the skeletal lineages independent of developmental stages (Table 1). There is enough reason to believe that all here reviewed putative multipotent stromal populations are comprised of cell mixtures that gain multipotency from heterogeneity rather than being one defined cell type. Another major caveat of most mouse SSC markers is the inability to translate them to humans (Figure 1). This has also become obvious when we identified a human SSC, which exhibits a different marker profile than the mouse SSC population we described. Investigating *bona fide* SSCs is crucial since the study of impure cell populations will keep on hampering the discovery of promising therapeutic approaches.

**TABLE 1:** Summary of selected markers proposed to enrich for mouse skeletal stem cells and multipotent stromal cells.

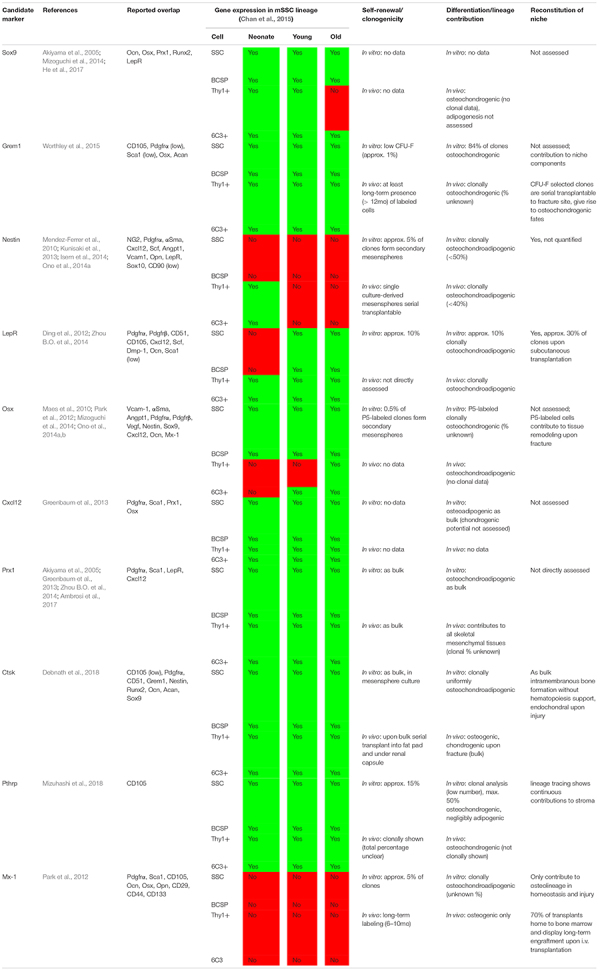

## Skeletal Stem Cell Development

Bone formation mainly occurs through two mechanisms: intramembranous and endochondral ossification, with the former describing direct osteogenesis from stromal cells, while the latter develops mineralized tissue through a cartilage template. As the primary form of bone formation in the appendicular skeleton, endochondral ossification is crucial for establishing the hematopoietic niche (Chan et al., 2009). Craniofacial bones arise from paraxial mesoderm and primitive neural crest following both, intramembranous and endochondral ossification routes. Excitingly, in a model of mandibular distraction, we could show that mSSCs can reverse to their early neural crest state in order to orchestrate directed regeneration (Ransom et al., 2018). Many proposed SSC markers of the cranium are distinct from the appendicular skeleton, which might be at least in part explained by their differential developmental origin. Axin2, for example, labels a stem cell pool throughout all stages of development and adulthood of mice which is virtually absent in long bones (Maruyama et al., 2016). These cells are also functionally distinct as they normally do not form chondrocytes and are less supportive of an ectopic hematopoietic niche when transplanted under the renal capsule. Together with Gli1-positive cells Axin2 is recognized as the best marker for cranial SSCs (Zhao et al., 2015). Gli1-expressing cells share characteristics with long bone BMSCs and are abundantly present along the whole craniofacial bone gaps, called sutures, while Axin2-positive stromal cells are restricted to the midline of these sutures. Both cell types are thought to contribute to growth and regeneration. These and other stromal cell markers of the cranium have been more comprehensively reviewed elsewhere (Doro et al., 2017).

Genetic lineage tracing in mice has allowed an unperturbed look into the developmental paths of SSCs. In the early embryo, bone-forming cells originate from the adjacent perichondrium of the cartilage anlage and migrate into the bone marrow cavity where they transiently undergo osteogenesis before disappearing in early postnatal life (Maes et al., 2010; Mizoguchi et al., 2014; Ono et al., 2014b). These cells have been tracked through Osterix-CreERt and a Col1(3.2kb)-CreERt lines. While Osx-expressing cells are enriched for cell populations with high CFU-F shortly after birth, they show a limited capacity to contribute to mature bone, cartilage, and fat lineages during adulthood. It has been implied that perinatal Osx-positive cells are a source of LepR^+^ cells which are the main origin of bone formed in adult mice (Liu Y. et al., 2013; Mizoguchi et al., 2014; Ono et al., 2014a).

Initial cartilage templates for bone formation are derived from Sox9-expressing progenitors, which are essential for skeletal growth and if traced from early embryonic stages mark the entity of osteogenic, chondrogenic, adipogenic, and stromal cell types including CAR cells of the adult bone marrow (Akiyama et al., 2005). They have also been assigned important roles as pSSCs during fracture healing (He et al., 2017). CD105 is an early marker of skeletal lineage commitment and can be detected as early as E13 (Chan et al., 2013). E14.5 limbs digested to single-cell dilution can re-create an ectopic bone marrow niche when transplanted under the renal capsule of adult mice, inferring that a SSC is present before blood vessel network formation (Chan et al., 2009). In support, E13.5 Grem1-Cre traces almost the entire embryonic mesenchyme and primary spongosia, which in combination with the metaphyseal anatomical localization of adulthood Grem1-expressing cells further strengthens the fact that a putative stem cell population has an inherent chondrogenic phenotype. This also fits with a theory in favor of the claim that the hypertrophic chondrogenic zone contains non-apoptotic chondrocytes acting as stem cells and when marked by Col10-Cre or Col10-CreERt can be followed to differentiated osteoblasts (Yang et al., 2014; Zhou X. et al., 2014; Mizuhashi et al., 2018). Lastly, Newton et al. (2019) recently reported the formation of a distinct stem cell niche in the early postnatal growth plate with a hierarchical structure important for tissue renewal, backing the presence of non-perivascular SSCs at this specific site.

The establishment of a blood vessel network in the newly formed bone marrow cavity from around E14.5 allows the formation of perivascular niches which are essential for the establishment of hematopoiesis. HSCs are present as early as E17.5 in long bones of mice and co-emerge with 6C3-supportive stroma and LepR-expressing cells, underscoring the necessity of the existence of a proper niche (Chan et al., 2013; Zhou B.O. et al., 2014). Furthermore, LepR-Cre targeted cells contribute to all osteogenic, chondrogenic, and adipogenic lineages in the adult skeleton (Ding et al., 2012; Zhou B.O. et al., 2014). They, however, very likely do not label a unique SSC population, due to their ubiquitous expression, enforcing the need for more specific SSC tracers.

In summary, Grem1-positive and mSSC cells are the first stem cell populations to appear during skeletal development and can be found throughout adulthood with contributions to osteogenic and chondrogenic cells for homeostasis and fracture repair. Perisinusoidal multipotent cell types arise during late embryogenesis and are a source for bone, cartilage, marrow fat. The exact proportions of the output to skeletal tissues and functional differences of these two cell types warrant further investigations in the future.

## Functional Characteristics of the Skeletal Stem Cell Lineage

Seminal findings of clonogenic fibroblasts in the bone stroma have early on described the capacity of these cells to form an ectopic bone marrow compartment complete with host-derived hematopoietic tissue. This entails the interaction of complex three-dimensional architecture with controlled expression of niche factors guiding cell fates. SSCs and bone “mesenchymal stromal cells” give rise to various stroma populations with distinct contributions to the tissue, i.e., bone formation, chondrocyte replenishment, bone marrow adipocytes accrual, and hematopoiesis-support, which all play their part in orchestrating skeletal organ function. The interaction of skeletal lineage cells with the blood-forming compartment has since been shown to be of uttermost importance. Aberrant changes in those tightly regulated networks result in stem cell lineage-based impairments which are potentially a cause for many bone-related diseases. Skewing SSC fates into desired lineages might hold great potential for translational approaches beyond skeletal tissue maintenance and repair; and toward treatments for hematological malignancies. Current therapeutic strategies involving “mesenchymal stromal cells” are mostly unsuccessful or moderate in effect as they largely rely on their immune-modulatory effects. Working with defined and controllable stem cell populations will pave the way to new, more effective approaches.

### Hematopoietic Niche Regulation

The conception of a stem cell niche, as envisioned by Schofield, set the stage for investigations into the regulation of HSCs by other cell types (Schofield, 1978; Kfoury and Scadden, 2015). Osteoprogenitor and osteoblastic cells were the first cells thought to have an important function in controlling hematopoiesis (Taichman et al., 2001). Later, transplantation experiments revealed that hematopoietic stem and progenitor cells (HSPCs) homed to perivascular and endosteal sites where they self-renewed and differentiated (Kiel et al., 2005; Sipkins et al., 2005; Lo Celso et al., 2009), an observation in close alignment with the primary localization of multipotent stromal cells. Cre-transgene mediated ablation of the osteoblastic compartment or expressed regulatory genes thereof, e.g., Scf, Cxcl12, subsequently confirmed that the osteogenic lineage did not markedly interact with hematopoiesis (Kiel et al., 2007). In contrast, the significance of more immature cell types, in particular labeled by Prx1, LepR, or Nestin, could be shown by targeted depletion of HSPC maintenance genes from the same (Mendez-Ferrer et al., 2010; Greenbaum et al., 2013; Zhou B.O. et al., 2014). Endothelial cell subsets are equally important for hematopoiesis which has been reported to be through Notch signaling as well as their permeability regulating reactive oxygen species exposure on HSPCs which affects features such as migration, survival, differentiation, and long-term repopulation capability (Itkin et al., 2016; Kusumbe et al., 2016). On top of that, the endothelium mediates important niche functions by directly acting on stromal cells involved with HSC control (Kusumbe et al., 2014). Lastly, bone marrow adipose tissue controls niche behavior in a context-dependent manner. Marrow adipocyte accumulation during obesity and aging impair hematopoiesis (Naveiras et al., 2009; Ambrosi et al., 2017), while adipocyte-derived SCF has been implied to promote regeneration after irradiation damage (Zhou et al., 2017).

Hematopoietic stem and progenitor cells and downstream lineages express many cognate receptors for factors produced by the stromal cell compartment but produce cytokines allowing two-way communication alike (Chan et al., 2015). Reverse regulation of HSCs to stromal cells is an intriguing idea and would give credit to the complexity of the niche model, a concept that needs to be looked at more intensively. Interestingly, metabolic sensing might be mostly restricted to the stromal compartment, since HSPCs express no common receptors involved in this process, e.g., LepR, PTH1R. Overall, this supports a model in which the skeletal niche tightly controls HSC maintenance and output.

It has to be noted that most experiments studying the interaction of blood stem cells with the stromal cell compartment are conducted with cytotoxic pre-conditioning, e.g., irradiation, and the use of unspecific Cre-driver transgenes. This most definitely affects experimental readouts, supported by divergent results for studies under homeostatic conditions and after transplant. The development of more cell type-restrictive transgenic mice and novel methods for niche interrogations are constantly adding to this active field of research and will shed more light on the regulation of HSCs by specific cell populations.

### Factors Impacting Lineage Fate

Diverse signals can direct lineage fates of multipotent BMSCs, which has consequences for the hematopoietic niche and is equally important for the balance of bone formation and resorption. Metabolic and age-related changes can favor adipogenic over osteochondrogenic differentiation paths in multipotent stromal cells, thereby impeding bone homeostasis (Yue et al., 2016; Ambrosi and Schulz, 2017; Tencerova et al., 2019). It remains to be shown if “our” *bona fide* SSCs, which under homeostatic and injury conditions do not contribute to the adipocytic lineage, are also able to acquire adipogenic fates during obesity, aging, or disease. The intercommunication between bone and other tissues has been established and is to a large extent mediated through stem cells (Idelevich and Baron, 2018). For instance, gut microbiota can control circulating IGF1 levels to induce bone formation (Yan et al., 2016). Moreover, a central signaling axis from the brain might be governing activity and lineage decisions of SSCs opening up a whole new research area (Herber et al., 2019).

Cartilage degeneration causing arthritis is one of the major health burdens of our society. It is unclear why articular cartilage at bone surfaces fails to be repaired. Currently, it is thought to be because of the avascular and structural nature of cartilage. Alternatively, it could be implied that SSCs lose their intrinsically defaulted chondrogenic fate displayed during growth and are skewed toward osteogenic and fibroblastic phenotypes. Strategies to target SSCs for stimulated cartilage output hold great promise for future treatments of this disease. Notably, we were able to show that BMP2 alone promotes expansion to an osteogenic fate of SSCs, while an additional VEGF-inhibitor application results in abundant cartilage formation (Chan et al., 2015). SSC lineages are main sources of these two factors accentuating that they are in part regulating their own niche by paracrine signaling, which can be modulated according to prevalent cues from the surroundings. Simultaneously, extrinsic alterations interfering with the niche guide aberrant lineage output. This is exemplified by the observation that cells labeled by PTHrP-CreERt directly differentiate into Col1a1(2.3kb)-GFP^+^ osteoblasts upon micro-perforation injury (Mizuhashi et al., 2018), losing their controlled fate upon niche interruption. Single multipotent BMSCs are reliant on a supportive niche in the form of feeder cells for engraftment and the ability to efficiently proliferate and differentiate in *in vitro* assays, respectively, underlining the necessity of a supportive niche (Chan et al., 2013; Ambrosi et al., 2017). Altogether these findings substantiate the notion that microenvironmental crosstalk and integrity is essential for proper stem cell function.

Age-related decline of tissue function is associated with bone loss and increased fracture risk, promoted by an osteoporotic status (Burge et al., 2007). Several reasons for stem cell aging have been described, i.e., telomere attrition, genomic instability, epigenetic alterations, loss of proteostasis, cellular exhaustion/senescence, and mitochondrial dysfunction/oxidative stress (Lopez-Otin et al., 2013). *In vivo* findings on the mechanism of compartmental aging of skeletal stromal cells is limited (Ganguly et al., 2017). Recent work has suggested that inflammation drives intrinsic adaptations in LepR-positive stromal cells with advancing age that decrease numbers and display loss of osteogenic capacity leading to impaired regeneration (Yukata et al., 2014; Josephson et al., 2019). Similarly, perivascular SSCs lose their osteochondrogenic differentiation ability while maintaining adipocyte formation *in vitro* and preferentially generate adipogenic cell types *in vivo* (Ambrosi et al., 2017). Using advanced stem cell characterization techniques, we could also demonstrate that metabolic disease such as diabetes mellitus represses expression of Indian Hedgehog (IHH) in SSCs, vested on high exposure of circulating tumor necrosis factor-alpha (TNFα), impairing bone repair. Pharmacological re-exposure of SSCs to exogenous IHH could rescue diabetic bone healing (Tevlin et al., 2017).

Furthermore, compositional changes of the bone stroma during aging might disrupt the hematopoietic niche causing the pronounced skewing to myeloid fates and mediating expansion of the HSPC pool. A consequence of the niche dysregulated in this manner is the increased production of bone-resorbing osteoclasts and simultaneous decrease of osteochondrogenic tissue formation, additionally corroborating loss of skeletal mass. RANKL and MCSF are essential and sufficient to drive osteoclast maturation, survival, and differentiation from the monocytic lineage (Cenci et al., 2000). While mature osteocytes have long been thought to be the main source of pro-resorbing cytokines (Nakashima et al., 2011), other findings favor the importance of more immature stromal cell populations as a source for osteoclastic factors (Cao et al., 2005; Tikhonova et al., 2019). Recently, it was demonstrated that RANKL reverse signaling from osteoclasts to osteoblasts can also stimulate bone formation (Ikebuchi et al., 2018). Follow-up investigations will have to unravel the implications of this mechanism as well as the exact role of stromal cell-derived signals for age-related processes. Finally, the possibility of a switch of skeletal lineages to an abundantly fibroblastic phenotype accompanying modifications of the extracellular matrix accelerating aging symptoms has prevailed to be an understudied direction of research. Targeting stem cell-sensed inflammation, reciprocal signaling with the hematopoietic niche, and cellular senescence comprise promising interventions for beneficial effects on bone health (Baker et al., 2011; Campisi, 2013).

## Caveats of Skeletal Stem Cell Biology

The field of SSC research has made fundamental progress over the last 60 years driven by technological advancements and the evolving potential of stem cell-based therapeutics. Results describing prospective SSC populations and mechanistic backgrounds of their function and regulation have been pleiotropic. Although we have learned important lessons from basic science, we are far from effective translational strategies. Reasons can be found in the examination of incompletely purified stem cell populations, discordant use of definitions and terminology as well as overinterpretation and conclusions drawn from flawed experimental models.

A major caveat of BMSC populations is their varying heterogeneity in terms of the actual percentage of cells with *bona fide* stem cell characteristics which in this new research era we are now able to address with single-cell genomics. It also will be compelling to see if highly purified cell populations such as the mSSC can undergo “clonal skeletogenesis”, meaning that single cells acquire a fitness advantage over others, may it be through genetic mutations or local stimuli. This will be particularly interesting from the perspective of skeletal maintenance, aging, and disease, comparable to what has been shown for HSCs (Jaiswal et al., 2014, 2017). Through the recent demonstration of mouse and human SSCs we are now in the position to interrogate inter-species and inter-bone site differences of SSCs, providing us with a platform for the delineation of clues on the intrinsic determinants of skeletal size and shape. We envision this to be harnessed for novel therapeutic strategies.

### Pitfalls of Skeletal Stem Cell Characteristics Assessment

The matter of heterogeneity warrants a re-examination of results by Owen and Friedenstein; that is the varying differentiation potential of CFU-F cells derived from marrow aspirates and their depiction as a non-homogeneous cell mixture manipulatable for multiple directions of differentiation. For a long time, the lack of well-defined functional assays similar to what is available for HSCs allowed only retrospective analysis of stem cell characteristics. The nature of plastic adherence cultures has strongly limited the analysis to a few primitive cells, ignoring the fact that quiescent stem cells might not readily attach in these assays. The underestimation and inaccurate characterization of stem cell-like cells become obvious when comparing cultures of the same cell type with or without feeder layer. Single feeder layer-supported candidate SSCs show higher survival and tri-lineage potential *in vitro* (Ambrosi et al., 2017). Two-dimensional culture limitations are partially overcome by mesensphere cultures which nevertheless still have to deal with the exposure to random factors compared to their endogenous niche. Fetal calf serum is an essential additive for stromal cell culture, however, is of unknown composition (e.g., hormones, growth factors, steroids, etc.) depending on lot number, which very likely affects properties of cultured cells and makes it hard to compare or reproduce results between laboratories. The ISCT has agreed on a marker panel expression for multipotent cultured stromal cell populations, which might hold true for the *in vitro* setting but is not transferable to the physiological setting, as often done; and more importantly characterizes fibroblastic instead of stem cell characteristics (Dominici et al., 2006). Cell surface epitope expression is prone to change depending on isolation method, culture conditions, and cell-detachment methods (discussed earlier).

Functional analyses are usually conducted on a selected number of highly proliferative clones which are passaged and treated with artificial cues for acquiring putative cell fates *in vitro*. There is also no common agreement on how to interpret the maturation stage of stem and progenitor cells by *in vitro* differentiation assays. While robust differentiation toward induced fates is mostly understood as multipotency originating from a stem cell, some laboratories have argued that populations with less pronounced differentiation phenotypes might be indicative of a more immature hierarchical state (Merrick et al., 2019).

Flow cytometry allows the prospective isolation of putative SSC populations that can be investigated for stem cell properties by direct transplantation into an *in vivo* setting without prior culture. Preparation of FACS samples entails the use of mechanical and enzymatic digestion, which also brings about risks for cell loss and changes of cell epitopes. Transcriptomic comparisons of bulk tissue versus sorted viable populations using deconvolution techniques such as CIBERSORT can help to identify potential cell types that may be only inefficiently isolated by FACS (Newman et al., 2015). Yet, it can be assumed that isolation of cells by surface marker expression is superior to selective and artificially skewed adherence cultures. It is also important to control if antibody panels for distinct cell types are inclusive of the same cells when working with treatment, disease, or aged cohort samples. The same applies when examining stromal cells from different skeletal sites. In this context, reporting of cell type abundance is highly ambiguous, since cellular composition, preparation method, and presentation of analysis often vary.

Taken together, the scientific community should refrain from defining “stem cells” based on their *in vitro* behavior. Rigorous *in vivo* testing of prospective cell populations will give a better understanding of true SSCs. Proof for the translatability of findings from mice to humans should always be aimed for, specifically, since many stem cell markers are not conserved between the two species, a prominent example being the cell surface configuration of mouse and human SSCs (Chan et al., 2015, 2018). The development of novel and more sophisticated techniques are needed to enable interrogations of SSCs *in situ* across species.

### Limitations of Transgenic Mouse Models

One means of looking at stem cell dynamics without perturbations is with the help of transgenic mice. Unfortunately, the use of gene promoters is inherently limited and biased (Mendez-Ferrer et al., 2015). No single genetic marker can target a pure skeletal or stromal stem cell population to date. Fluorescent reporter protein presence under control of a target gene can be misleading and not reflective of the actual expression. For instance, minimal thresholds of transcriptional activity for fluorescence protein expression and detection are varying depending on gene construct and reporter protein used. Immature stromal cells also tend to express high levels of genes necessary for differentiation, which not necessarily means that they have already committed to a lineage which would be represented by the investigated marker. Lastly, fluorescent protein retention after gene expression has ceased also gives a false readout.

The gold standard for lineage tracing is the Cre-LoxP system. Although very efficient for a broad majority of tissues it comes with major limitations for bone tissue (Elefteriou and Yang, 2011). Potential causes have been implied to result from the chromosomal location of floxed alleles, the distance between LoxP sites, and cell-specific Cre-activity (Liu J. et al., 2013). A number of Cre-lines targeting common stromal cell markers (PDGFRα, Prx1, Osx) have been demonstrated to only incompletely recombine in the bone marrow (Krueger et al., 2014; Mizoguchi et al., 2014; Zhou B.O. et al., 2014; Ambrosi et al., 2017). To the opposite, some Cre-reporters show fluctuating levels of leakage in cells not expressing the transgene. Different models for the same reporter gene have yielded divergent results. Reporter mice for the osteogenic transcription factor Osterix, for example, label only committed osteoblastic cells in an earlier model while also recombining in stromal cell types in newer inducible versions (Madisen et al., 2010; Maes et al., 2010; Liu Y. et al., 2013). Evidence that Cre-expression alone can affect skeletal maturation has also been reported in an Osx-Cre model, necessitating a rigorous assessment of each transgenic line (Wang et al., 2015).

Investigations of functional effects mediated by Cre-drivers often ignore quantitative and qualitative off-target effects. In the context of constitutive Cre-expression, compensatory effects for the lack of a deleted target gene have to be considered, especially during development. Cre-driven gene deletions are also often rather inefficient, reflected in the moderate phenotypes contrary to the expectation. Timepoint-specific deletions by inducible Cre-ERt models are many times hard to interpret as they require tamoxifen administration which has been shown to have side effects, even more pronounced when applied long-term. This has been partially overcome with the development of Cre-ERt2 models, which require up to ten times lower amounts of tamoxifen administration. Possible side issues should also be accounted for when using other activators of genetic modifications such as doxycycline, tetracycline, and diphtheria toxin. Drawbacks of single markers used to characterize stem cell populations are obvious. Prominent examples of Cre-lines misinterpreted as selective multipotent stromal cell markers include Mx-1, Nestin, NG2, and LepR (Ulyanova et al., 2007; Park et al., 2012; Mizoguchi et al., 2014; Zhou B.O. et al., 2014). Aside the broad spectrum of stromal cell types they select for, they additionally span hematopoietic and endothelial cell populations. When using these Cre-models for directed deletion of genes in bone populations it is also important to consider functional readouts of indirect origin, in these cases of neural origin, especially on the note that brain-derived factors affect bone physiology.

Thus, data has to be interpreted with all these potential pitfalls in mind. Appropriate controls for background signal determination are important. Transgene reporter expressions should always be validated in multiple ways, e.g., FACS, qPCR, or histology. In general, it is advisable to combine fluorescence protein expression with additional antibodies against surface proteins. This is allowing the investigation of more purified cell types, which couldn’t be looked at without fluorescence-tagging of non-surface markers. Overall, comprehensive approaches combining rigorous validation of designated cells and functional testing of stem cell hallmarks in *in vivo* assays are necessary. This will also help to lead the way to the identification and development of more specific SSC Cre-models.

## Conclusion and Future Directions

The field of SSC research holds enormous promise for a paradigm shift in the treatment of bone-related diseases and the understanding of pathogenetic mechanisms. In order to accomplish meaningful translational results, the research community will be better served if there are updated guidelines for the definition and nomenclature of SSCs; in the spirit of the ISCT publication (Dominici et al., 2006). However, it is necessary to amend the ISCT guidelines as they continue to build on the initial problematic definition of “MSCs” (Caplan, 1991). Constituting a revised definition will have to be based on the latest technological advancements that have granted a better understanding of the function and regulation of single stem and progenitor cell types. Integrating the state-of-the-art knowledge and available resources of bone stem cell biology to us, we have come to suggest minimal criteria and terms for defined *bona fide* stem cells of the skeleton.

### Proposed Bona Fide SSC Criteria

*In vitro* methods to derive true tissue-specific stem cells, expand patient-derived stem cells, and protocols to differentiate them into functional tissues are highly important topics in stem cell research and bioengineering. However, *in vitro* cell culture experiments have limited value in assessing *in vivo* cell behavior and should not exclusively be considered as evidence in the determination of stem cell activity. This has been extensively shown for bone marrow-derived stromal cells, which display differentiation capacity beyond mesodermal fates *in vitro* but are much more restricted *in vivo* (Bianco et al., 2006). SSCs should be able to self-renew, give rise to progeny of more restricted cell fate, and differentiate at least into osteoblasts/osteocytes and chondrocytes, all on the clonal level *in vivo* without prior *in vitro* culture (Bianco et al., 2008). Self-renewal should be considered maintaining the original phenotype while simultaneously giving rise to cell populations and stroma of more mature states which together are able to reconstitute an entire stem cell compartment *in vivo* able to recruit active hematopoiesis; and not be confused with sustained growth and differentiation capacity in culture.

These properties should be observed upon transplantation of freshly isolated cells into the endogenous environment but can also be assessed when transplanted into ectopic sites, e.g., renal capsule, adipose tissue, subcutaneous; to determine their intrinsic skeletogenic potential. It remains to be investigated, however, if SSCs might suffer from exhaustion much faster than HSCs because the inherent tissue turnover is manifolds slower. In contrast to HSCs, which can home to their bone marrow niches after intravenous injections, SSCs have to be transplanted into the desired tissue and, more importantly, need stromal cell support in order to engraft at the single-cell level (Chan et al., 2013). Functionally, as also exemplified by HSC approaches, proof of substantial contributions to skeletal tissue during homeostasis and/or injury should be provided for candidate cell populations, may it be through lineage tracing or long-term tracking of donor cells after transplantation.

Based on the possibility that multiple stem cell types exist within the bone and the observation that SSCs defined by us and others do not make adipocytes, tri-lineage differentiation capacity should not be a prerequisite for stem cells. Likewise, at this point, we are not able to define common markers for all bone-resident stem cells, except that they are most likely of non-hematopoietic (CD45^–^) and non-endothelial (CD31^–^) origin. A major challenge will be to discern the existence of SSCs at multiple anatomical sites throughout development, adulthood, and repair. The constant dynamic adaptations of skeletal tissue will require a careful characterization of all potential stem cell niches. Currently, it seems likely that discrete microenvironments of the same bone harbor distinct stem cell populations with similar properties but dedicated physiological functions depending on the localization.

### SSC Terminology From Here on Onward

While considering the difficulties in altering a widely engrained nomenclature and acknowledging the improbability that the general usage of “MSC” will be discarded any time soon (Caplan, 2017), we would still like to propose a couple of specifications to address some of the more confusing and negative connotations of the term. The name “mesenchymal stem cell” is chronically used in an inappropriate fashion to describe a cell population with stem cell features as a whole but high heterogeneity on the single-cell level and, therefore, should be abandoned. *Bona fide* SSCs can be considered bone-resident stromal stem cells. Similarly, any other connective tissue contains a comparable, but specific stem cell type. If minimal criteria of stem cell state have not been properly investigated, cell populations from these tissues should be referred to as “multipotent stromal cells,” indicative of mixed stromal cell types of the corresponding source tissue. “SSC” should be reserved for the highly selective and homogeneous cell population responsible for bone organ development, growth, and regeneration/repair only. Contingent upon the anatomical microenvironment these cells reside in, SSCs could be further specified, e.g., growth plate SSCs (gpSSC), periosteal SSCs (pSSCs), etc.

Connective tissues harbor different amounts of stem cell types depending on the lineages required for proper organ function. The terminology of stem cells should be reflective of the tissue origin and contributions, as successfully implemented with SSCs. Evidence for a second distinct perivascular stem cell in long bones, which seems to be predominantly involved in hematopoietic maintenance and niche support, and which is more representative of classic “MSCs” when assayed *in vitro* leads us to propose the classification of a perivascular SSC (pvSSC), based on its localization and properties. Future experiments will have to shed more light on the characteristics and functions of subpopulations of SSCs and will have to further evolve terminology applied to these cell types. Eventually the scientific community should establish a timely and accurate cell atlas for reporting new skeletal lineages within the context of previously discovered lineages in real-time.

## Author Contributions

TA, ML, and CC wrote and edited the manuscript. TA prepared the figure.

## Conflict of Interest Statement

The authors declare that the research was conducted in the absence of any commercial or financial relationships that could be construed as a potential conflict of interest.
